# Analysis of trends and usage of ICD-10-CM discharge diagnosis codes for poisonings by fentanyl, tramadol, and other synthetic narcotics in emergency department data

**DOI:** 10.1016/j.abrep.2022.100464

**Published:** 2022-10-20

**Authors:** Shannon M. Casillas, Lawrence Scholl, Desiree Mustaquim, Alana Vivolo-Kantor

**Affiliations:** National Center for Injury Prevention and Control, Centers for Disease Control and Prevention, Atlanta, GA 30341, USA

**Keywords:** Overdose, Opioid, Fentanyl, Drug, Epidemiology, Surveillance, AMPC, average monthly percent change, CDC, Centers for Disease Control and Prevention, CI, confidence interval, ED, emergency department, ESSENCE, Electronic Surveillance System for the Early Notification of Community-based Epidemics, IMF, illicitly manufactured fentanyl, MPC, monthly percent change, NSSP, National Syndromic Surveillance Program, TOM, Traceable Opioid Material

## Abstract

Synthetic opioids, including illicitly manufactured fentanyls, are driving recent increases in US overdose deaths. Beginning October 2020, the International Classification of Diseases, 10th revision, Clinical Modification (ICD-10-CM) code for poisonings involving synthetic narcotics (T40.4X) was split into three codes: fentanyl (T40.41), tramadol (T40.42), and other synthetic narcotics (T40.49). Emergency department data from October 2019–September 2021 in the Centers for Disease Control and Prevention’s National Syndromic Surveillance Program BioSense platform were queried for synthetic opioid codes in the chief complaint and discharge diagnosis fields. Trend analyses assessed average monthly percent change overall and by sex and age. Emergency department visits for overdoses involving synthetic narcotics increased on average 3.2 % each month before the code split and 4.8 % after. Visits with fentanyl codes drove this increase after the split, accounting for most visits among males, females, and every age group except ≥ 65 years. The average monthly percent increase for ED visits for fentanyl-involved overdoses was greater than for all synthetic narcotics combined (i.e., T40.41, T40.42, and/or T40.49), suggesting that the old code (T40.4X) masked the full extent of the increase in ED visits for fentanyl overdoses. Usage of these new codes can improve tracking of non-fatal synthetic opioid overdose trends.

## Highlights

• More specific ICD-10-CM synthetic opioid poisoning codes implemented October 2020.
• A single code was split into three new codes, including one for fentanyl poisoning.
• Usage and trends of the new codes were explored in emergency department data.
• Emergency department visits for synthetic opioid overdoses increased significantly.
• 76% of visits for synthetic opioid overdoses were coded as involving fentanyl.

## 1 Introduction

Synthetic opioids, specifically illicitly manufactured fentanyls (IMF) (including fentanyl analogs), continue to drive increases in drug overdose morbidity and mortality (Mattson et al., 2021, O'Donnell et al., 2021, Palamar et al., 2022, Rezaeiahari and Fairman, 2022) and may be displacing heroin in the illicit drug market in some communities (Pardo et al., 2019). The increasing availability, geographic dispersion, mixing or co-use of fentanyl with other drugs, and presence of IMF in counterfeit prescription pills resembling commonly misused prescription drugs underscore the need to not only better track fentanyl overdoses, specifically, but also to tailor prevention approaches (Centers for Disease Control and Prevention [CDC], 2020).

Tracking nonfatal drug overdoses involving fentanyl has been a noted challenge (Vivolo-Kantor et al., 2021), in part due to the lack of point of care testing. Traditional nonfatal injury surveillance systems have relied on standardized discharge diagnosis codes (i.e., International Classification of Diseases, 10th Revision, Clinical Modification [ICD-10-CM]) to assist in developing case definitions, yet up until October 2020, no specific fentanyl overdose ICD-10-CM code existed.^1^ A single T-code from the injury chapter—T40.4X (Poisoning by, adverse effect of and underdosing of other synthetic narcotics)—had been used as a proxy to estimate fentanyl overdoses; however, this code also included other manufactured narcotics such as tramadol and buprenorphine. The fiscal year 2021 ICD-10-CM specifications were released on October 1, 2020, and for the first time, included three new codes to replace the broad T40.4X code: poisoning by, adverse effect of and underdosing of fentanyl or fentanyl analogs (T40.41); poisoning by, adverse effect of and underdosing of tramadol (T40.42); and poisoning by, adverse effect of and underdosing of other synthetic narcotics (T40.49) (Centers for Medicare and Medicaid Services [CMS], 2020).

To date, no studies have explored the use of these new codes and the transition from the older ICD-10-CM code. The present study seeks to better understand monthly emergency department (ED) synthetic narcotic overdose trends overall and by sex and age group prior to and after the transition to the new codes on October 1, 2020 using data from the CDC National Syndromic Surveillance Program (NSSP).

## 2 Methods

Data from NSSP were used for analysis and queried from the BioSense platform using the Electronic Surveillance System for the Early Notification of Community-based Epidemics (ESSENCE) (CDC, 2021). NSSP is a collaboration of public health partners (e.g., the CDC, other federal, local and state health departments) to collect and analyze data from EDs and other healthcare settings (e.g., urgent care) in 50 states, DC, and Guam. Access to NSSP data was granted by participating states/jurisdictions. These data, captured in near real-time, can provide an early warning of possible overdose spikes and clusters (CDC, 2021). NSSP receives data from approximately 71 % of ED facilities in the US (CDC, 2021).

Monthly counts of ED visits that occurred from October 1, 2019–September 30, 2021^2^ were queried from all ED facilities in NSSP ESSENCE and visits were included if the chief complaint or discharge diagnosis fields contained the ICD-10-CM code for T40.4X, T40.41, T40.42, or T40.49 (CMS, 2020; National Center for Health Statistics, 2022); only codes utilized during the expected time period were used in analysis (i.e., T40.4X from October 1, 2019–September 30, 2020 and T40.41, T40.42, and T40.49 from October 1, 2020–September 30, 2021). All intents and encounters were included. To control for facility-level inconsistency in data reporting and quality throughout the study period, we restricted our analysis to data from facilities with a coefficient of variation ≤ 45 and a discharge data informative value of ≥ 75 % to account for the average weekly submission of visits containing informative diagnosis codes.^3^ This subset of included facilities came from 33 states and DC representing approximately 33 % of ED facilities in the US.^4^ Hereafter, year one refers to the period before the code split (October 1, 2019–September 30, 2020), and year two refers to the period after (October 1, 2020–September 30, 2021).

Analysis was conducted in R (R Core Team, 2019) and figures were created using the ggplot2 package v3.3.2 (Wickham, 2016). Counts were stratified by sex and age group (15–24, 25–34, 35–44, 45–54, 55–64, and ≥ 65 years); data for those < 15 years old were excluded from the stratified analysis due to small counts and data for figures were suppressed for months where counts were < 10. Joinpoint regression was used to assess the average monthly percent change (AMPC) with 95 % confidence intervals (CIs) in ED visits for each ICD-10-CM code (National Cancer Institute, Bethesda, MD); monthly percent change (MPC) estimates are footnoted where significant changes in trend (e.g., increase to decrease) were found.

## 3 Results

**Fig. 1:**
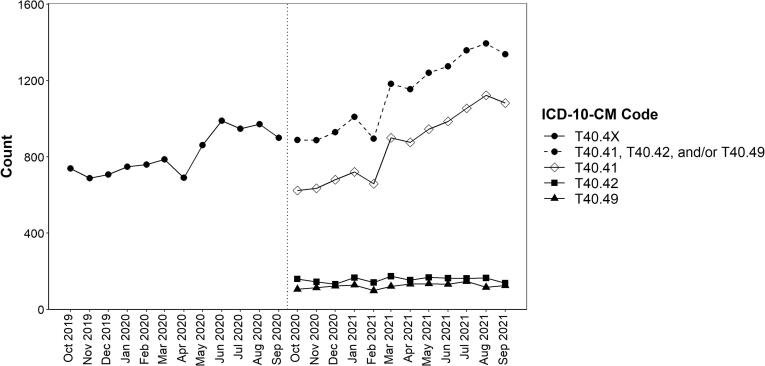
Monthly emergency department visits for overdoses involving synthetic narcotics (T40.4X), fentanyl (T40.41), tramadol (T40.42), and other synthetic narcotics (T40.49), National Syndromic Surveillance Program, 33 States and DC, October 2019–September 2021.

During year one, there were 9,787 ED visits for overdoses involving synthetic narcotics (T40.4X); during year two, there were 13,551 visits (T40.41, T40.42, and/or T40.49). During year two, approximately 76 % (n = 10,281) of ED visits for overdoses from synthetic narcotics involved fentanyl, 14 % (n = 1,859) involved tramadol, and 11 % involved other synthetic narcotics (n = 1,467) (these categories are not mutually exclusive, as some visits had multiple codes for synthetic narcotics). The overall number of ED visits for overdoses involving synthetic narcotics increased on average 3 % each month during year one (AMPC = 3.2; 95 % CI: 1.7, 4.7) and nearly 5 % each month during year two (AMPC = 4.8; 95 % CI: 3.6, 6.0) (Table 1 and Fig. 1). ED visits for overdoses involving fentanyl were the primary driver of the increases during year two, with an average 6 % increase each month (AMPC = 6.1; 95 % CI: 4.8, 7.4); no significant monthly changes were observed for overdose visits involving tramadol or other synthetic narcotics.

**Table 1:** Average monthly percent change in emergency department visits for overdoses involving synthetic narcotics (T40.4X), fentanyl (T40.41), tramadol (T40.42), and other synthetic narcotics (T40.49), overall, by patient sex, and by age, National Syndromic Surveillance Program, 33 States and DC, October 2019–September 2021.

		**Average Monthly Percent Change in ED Visits**§**(95 % CI)**			
	Year 1*	**Year 2**†			
	**T40.4X** **(All synthetic narcotics)**	**T40.41, T40.42, and/or T40.49** **(All synthetic narcotics)**	**T40.41 (Fentanyl)**	**T40.42 (Tramadol)**	**T40.49** **(Other synthetic narcotics)**
**Overall**	3.2 % (1.7, 4.7)	4.8 % (3.6, 6.0)	6.1 % (4.8, 7.4)	0.5 % (-1.3, 2.2)	1.6 % (-0.3, 3.5)
**Sex**					
Male	4.9 % (3.2, 6.6)	5.6 % (4.2, 6.9)	6.5 % (5.0, 7.9)	-0.5 % (-3.1, 2.3)	2.1 % (-0.3, 4.5)
Female	1.1 % (-0.6, 2.7)	3.4 % (1.9, 4.9)	5.1 % (3.2, 7.1)	1.0 % (-1.1, 3.1)	0.9 % (-1.4, 3.2)
**Age (years)**					
15–24	4.5 % (2.2, 6.9)	4.4 % (2.3, 6.5)	5.3 % (3.0, 7.5)	-5.1 % (-8.6, -1.4)	7.1 % (0.3, 14.4)
25–34	4.7 % (3.5, 5.9)	5.4 % (4.0, 6.7)	6.0 % (4.7, 7.3)	-0.3 % (-4.0, 3.6)	2.5 % (-2.1, 7.3)
35–44	4.6 % (0.1, 9.4)¶	6.9 % (4.8, 9.1)	8.0 % (5.9, 10.1)	0.6 % (-4.3, 5.7)	3.2 % (-1.6, 8.3)
45–54	1.2 % (-1.6, 4.1)	5.6 % (3.6, 7.6)	6.4 % (3.9, 9.0)	5.4 % (1.5, 9.5)	0.3 % (-3.5, 4.3)
55–64	3.8 % (1.5, 6.1)	3.4 % (1.6, 5.3)	5.5 % (2.9, 8.1)	0.3 % (-3.0, 3.7)	-1.3 % (-6.8, 4.5)
≥65	-2.3 % (-5.4, 0.9)	0.8 % (-1.3, 2.9)	1.2 % (-1.5, 3.9)	0.5 % (-2.1, 3.3)	5.5 % (-8.5, 21.6)¶

§ Average monthly percent change in ED visits for overdoses involving synthetic narcotics calculated using Joinpoint regression.

* Time period included October 1, 2019 through September 30, 2020.

† Time period included October 1, 2020 through September 30, 2021.

¶ The best fitting models for most analyses identified zero joins, with the exception of T40.4X among those 35–44 years old [decreased from October 2019–December 2019 (MPC = -13.4 (95 % CI: -34.1, 13.9)); increased from December 2019–September 2020 (MPC = 9.1 (95 % CI: 6.4, 11.9))] and T40.49 among those ≥ 65 years [increased from October 2020–December 2020 (MPC = 63.6 (95 % CI: -31.7, 292.1)); decreased December 2020–September 2021 (MPC = -4.3 (95 % CI: -11.7, 3.6))].

During year one, there was a significant increase in ED visits for overdoses involving synthetic narcotics only for males (AMPC = 4.9; 95 % CI: 3.2, 6.6) (Table 1 and Supplementary Figures). During year two, both males and females had significant increases in ED visits for overdoses involving synthetic narcotics; this was driven by overdoses involving fentanyl, with an average monthly percent increase of more than 6 % among males (AMPC = 6.5; 95 % CI: 5.0, 7.9) and 5 % among females (AMPC = 5.1; 95 % CI: 3.2, 7.1). Monthly ED visits involving tramadol and other synthetic narcotics remained stable among males and females.

During year one, ED visits for overdoses involving synthetic narcotics increased for persons aged 15–24, 25–34, 35–44, and 55–64 (Table 1 and Supplementary Figures). During year two, all age groups except those ≥ 65 years had significant increases in ED visits for overdoses involving synthetic narcotics; this finding was consistent with visits for overdoses involving fentanyl. Additionally, during year two ED visits for overdoses involving tramadol increased only among persons aged 45–54 years and decreased only among persons aged 15–24 years, and visits for overdoses involving other synthetic narcotics increased only among those aged 15–24 years. Among all age groups except those ≥ 65 years, overdoses involving fentanyl were the primary driver of ED visits for overdoses involving synthetic narcotics; among those ≥ 65 years old, visits for overdoses involving tramadol were higher than those for fentanyl.

## 4 Discussion

This is the first study to analyze the impact of new, more granular T40.4 ICD-10-CM codes for synthetic narcotics in national ED data. Overall, we found that nonfatal synthetic opioid overdoses increased throughout the study period, but increases were greater in year two; this is similar to previous research that found ED visits for nonfatal opioid overdoses were greater in 2020 compared to 2019 (Holland et al., 2021). Several factors could explain the greater increase in nonfatal synthetic opioid overdoses in year 2 of this analysis, including: 1) an expansion of IMFs into new geographic areas (e.g., Western U.S.) (Ciccarone, 2021); 2) disproportionate increases in the rate of overdose deaths among racial and ethnic minority groups from 2019 to 2020 (Friedman and Hansen, 2022, Kariisa et al., 2022); and 3) an acceleration in overdoses during the start of the COVID-19 pandemic, potentially due to increased social isolation and mental health impacts (Tanz et al., 2022). Based on findings after the code split, fentanyl was likely the primary driver of nonfatal synthetic opioid overdoses before the new codes were implemented for use in October 2020. This finding is consistent with data documenting mortality trends related to IMFs and other synthetic opioids (O’Donnell et al., 2017). After the code change, fentanyl was the most common synthetic opioid with significant increases overall, for both sexes and for all age groups except ≥ 65 years. The average monthly percent increase in ED visits for fentanyl-involved overdoses was greater than for all synthetic narcotics combined (i.e., T40.41, T40.42, and/or T40.49), suggesting that the old code (T40.4X) masked the full extent of the increase in ED visits for fentanyl overdoses. Our analyses demonstrated the potential usefulness of monitoring these codes in near real-time ED data systems to capture fentanyl-involved overdose visits.

This study was subject to at least three limitations. First, this analysis is not nationally representative. Approximately 33 % of ED facilities in the US were included as part of this analysis to ensure continuity in data coverage and completeness over the study period for assessment of trends; it is possible that included facilities are systematically different than those not included, thus biasing these findings. Second, this study relied on the use of diagnosis codes to categorize synthetic narcotic-involved overdoses treated in EDs; however, variability exists in how facilities determine assignment of diagnosis codes for patients. For example, point-of-care testing for fentanyl is inconsistently performed and confirmatory testing is limited (Bhalla, 2014); consequently, self-reports, bystander reports, and/or clinical presentations often are used to determine the drugs involved with overdoses. Unfortunately, reliance on this information can be problematic, leading to misclassification and underreporting of the drugs involved, especially if the patient was unaware the drug was adulterated (e.g., with fentanyl) (Ciccarone et al., 2017). More consistent and comprehensive drug testing methods in EDs can improve identification of substances involved in overdoses (Schwartz et al., 2022) and could help determine the reliability of diagnosis codes in accurately capturing the drug(s) involved. One available resource to improve synthetic opioid testing methods are Traceable Opioid Material (TOM) kits, which include reference materials for 250 synthetic opioid compounds, including 210 fentanyl analogs (CDC, 2022). Third, individuals are increasingly refusing transport to EDs following treatment by emergency medical services in the field (Bergstein et al., 2021, Slavova et al., 2020); thus, reliance on ED visits alone to assess trends in narcotic-involved overdoses may underestimate the true burden. Nevertheless, assessing the occurrence of these codes using ED data can lead to better and more timely tracking of fentanyl overdose trends, as demonstrated in this analysis.

Monitoring ED data for nonfatal fentanyl overdoses can help inform public health response and prevention efforts. Rapid identification of overdose spikes, including increases in ED visits for overdoses, can lead to increased and tailored naloxone distribution; initiation of treatment with medication for opioid use disorder; linkage to follow-up treatment and harm reduction services; and alerting of at-risk communities (Houry et al., 2018). Ultimately, having a reliable way to track fentanyl trends is critical, and these more granular ICD-10-CM codes, when backed by consistent and quality diagnostic methods, could be an effective way to do this.

## CRediT authorship contribution statement

**Shannon M. Casillas:** Conceptualization, Data curation, Formal analysis, Methodology, Project administration, Visualization. **Lawrence Scholl:** Conceptualization, Data curation, Validation, Methodology. **Desiree Mustaquim:** Conceptualization, Methodology. **Alana Vivolo-Kantor:** Conceptualization, Methodology.

## Declaration of Competing Interest

The authors declare that they have no known competing financial interests or personal relationships that could have appeared to influence the work reported in this paper.

## Footnotes

1 The ICD-10-CM classification includes the term “poisoning” to convey the mechanism and external cause of injury. In this study, the term “overdose” is used synonymously with “poisoning”.

2 The study period partially overlapped with the COVID-19 pandemic, which may have impacted care seeking behavior following an overdose; previous studies found an initial decline in ED visits overall following implementation of COVID-19 mitigation measures in March 2020 (Holland et al., 2021).

3 Data were queried on October 25, 2021 from NSSP ESSENCE. The coefficient of variation (CoV) metric was used to control for facility-level inconsistency in total ED visit data submissions to NSSP over the study period (e.g., inconsistency resulting from onboarding, interrupted or halted data feeds). Data from a given facility were included in the analysis if the facility had a coefficient of variation ≤45 across all ED visits submitted from the facility during the study period. The CoV is calculated by dividing the standard deviation by the overall mean for each facility’s daily volume of ED visits and is presented as a percentage of the mean (standard deviation divided by the mean multiplied by 100). Analysis also was restricted to data from facilities with ≥75% average weekly visits containing actual diagnosis codes during the study period; the discharge diagnosis informative metric in NSSP ESSENCE was applied to distinguish visits with actual diagnosis codes.

4 Facilities from the following states/jurisdictions were included: Alabama, Arizona, Arkansas, Colorado, Connecticut, Delaware, District of Columbia, Florida, Georgia, Illinois, Kansas, Kentucky, Louisiana, Maine, Maryland, Mississippi, Missouri, Montana, Nevada, New Jersey, New Mexico, New York, North Carolina, North Dakota, Oregon, Pennsylvania, Rhode Island, South Carolina, Tennessee, Utah, Virginia, Washington, West Virginia, and Wisconsin.
