# Supplementary material for: Analysis of trends and usage of ICD-10-CM discharge diagnosis codes for poisonings by fentanyl, tramadol, and other synthetic narcotics in emergency department data
Source: Addict Behav Rep. 2022 Oct 20;16:100464. doi: 10.1016/j.abrep.2022.100464 (PMC9661429; doi:10.1016/j.abrep.2022.100464)
Supplement: Supplementary data 1 [file mmc1.docx]

**Figure 2**. Monthly emergency department visits for overdoses involving synthetic narcotics (T40.4X), fentanyl (T40.41), tramadol (T40.42), and other synthetic narcotics (T40.49), by sex, National Syndromic Surveillance Program, 33 States and DC, October 2019–September 2021

**
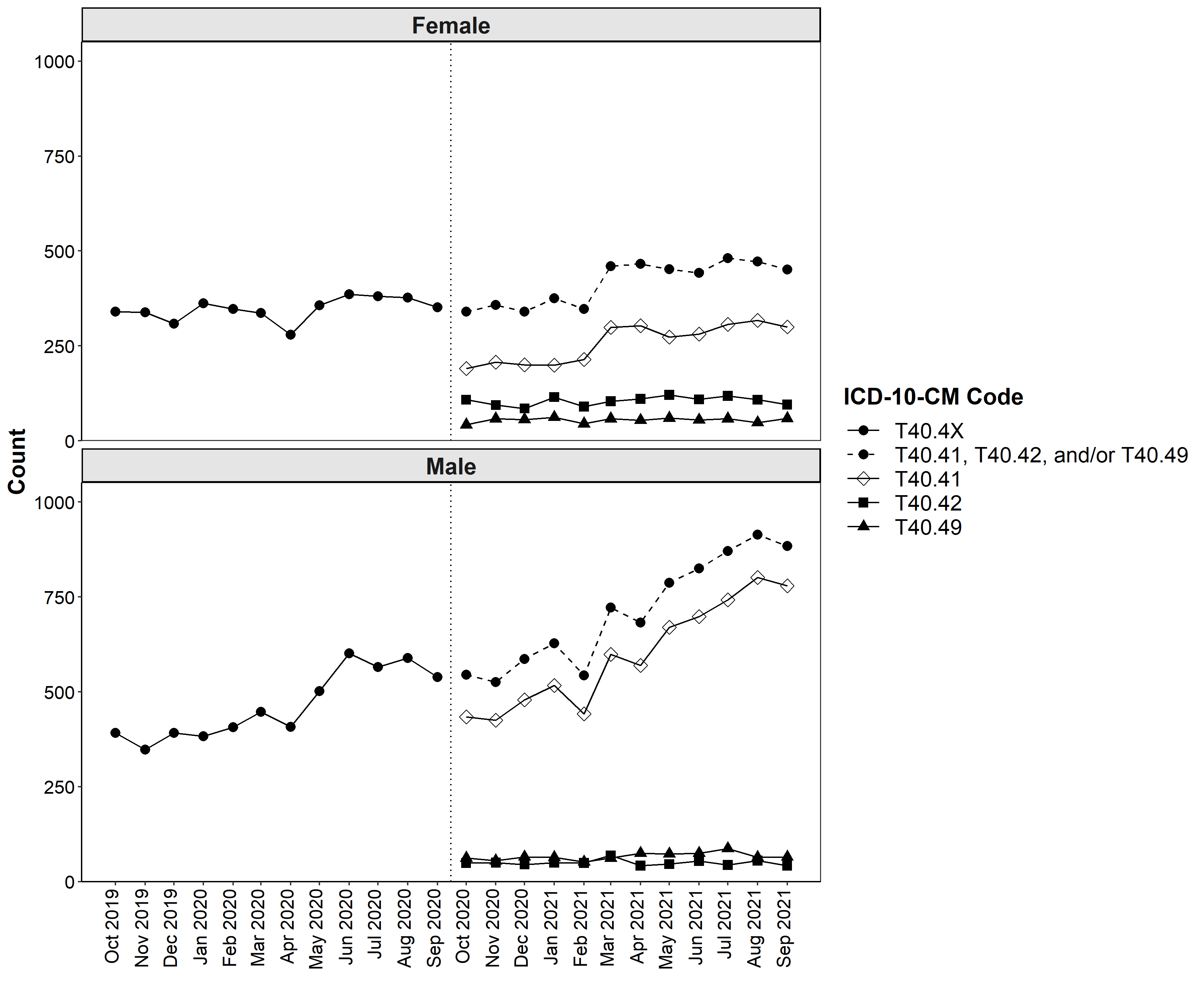
**

**Figure 3**. Monthly emergency department visits for overdoses involving synthetic narcotics (T40.4X), fentanyl (T40.41), tramadol (T40.42), and other synthetic narcotics (T40.49), by age group^‡‡^, National Syndromic Surveillance Program, 33 States and DC, October 2019–September 2021

**
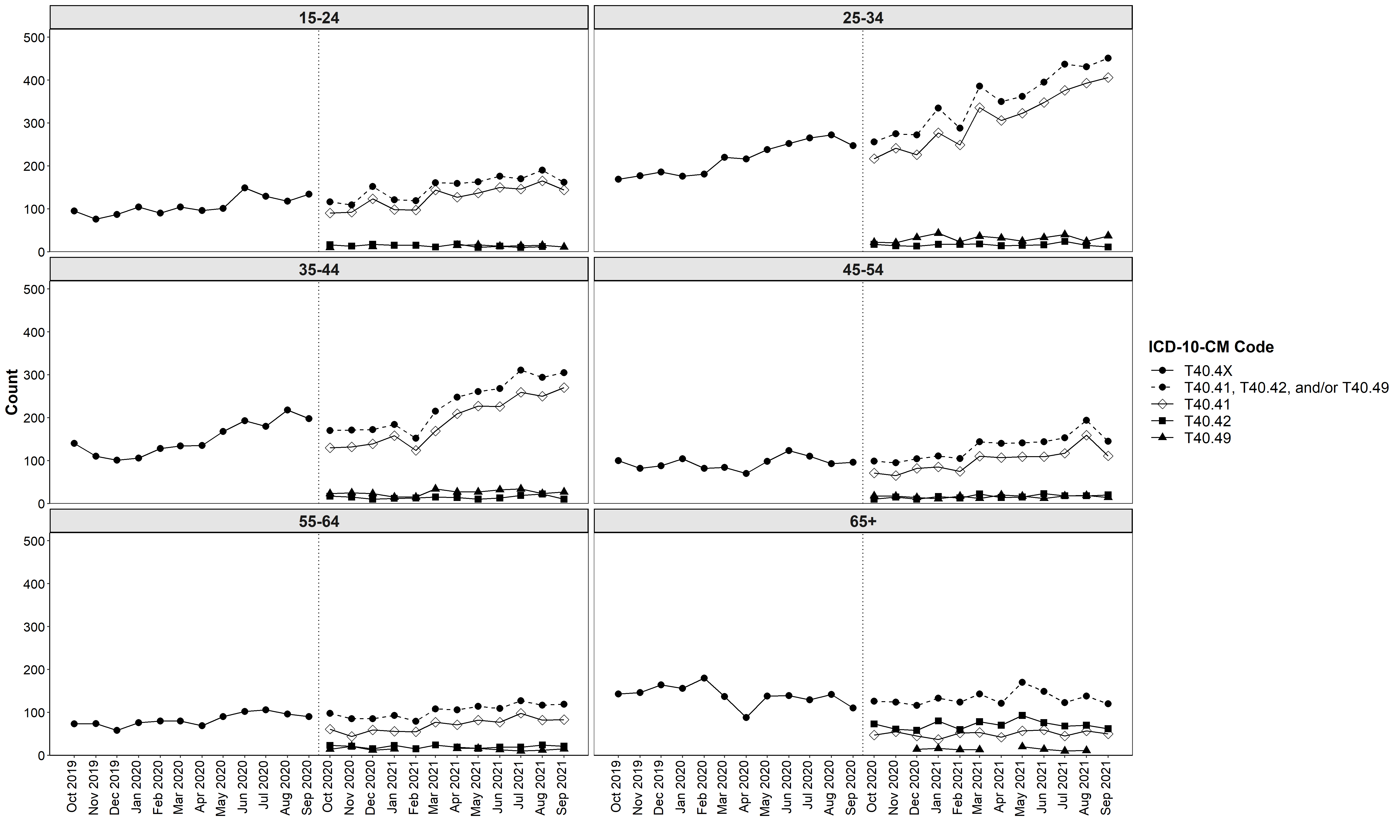
**

^‡‡^ Data for those <15 years old were excluded due to small counts. Data were suppressed for months where counts were <10.
